# Metabolism and Disposition of Aditoprim in Swine, Broilers, Carp and Rats

**DOI:** 10.1038/srep20370

**Published:** 2016-02-03

**Authors:** Liye Wang, Lingli Huang, Yuanhu Pan, Kamil Kuča, Blanka Klímová, Qinghua Wu, Shuyu Xie, Ijaz Ahmad, Dongmei Chen, Yanfei Tao, Dan Wan, Zhenli Liu, Zonghui Yuan

**Affiliations:** 1 National Reference Laboratory of Veterinary Drug Residues and MAO Key Laboratory for Detection of Veterinary Drug Residues, Wuhan, Hubei 430070, China; 2 MOA Laboratory for Risk Assessment of Quality and Safety of Livestock and Poultry Products, Wuhan, Hubei 430070, China; 3 Hubei Collaborative Innovation Center for Animal Nutrition and Feed Safety, Huazhong Agricultural University, Wuhan, Hubei 430070, China; 4 Center for Basic and Applied Research, Faculty of Informatics and Management, University of Hradec Kralove, Hradec Kralove, Czech Republic; 5 Biomedical Research Center, University Hospital Hradec Kralove, Hradec Kralove, Czech Republic; 6 College of Life Science, Yangtze University, Jingzhou 434025, China; 7 Hunan Provincial Engineering Research Center for Healthy Livestock and Poultry Production, Key Laboratory of Agro-ecological Processes in Subtropical Region, Institute of Subtropical Agriculture, Chinese Academy of Sciences, Changsha, 410125, China

## Abstract

Aditoprim (ADP) is a newly developed antibacterial agent in veterinary medicine. The metabolism and disposition of ADP in swine, broilers, carp and rats were investigated by using a radio tracer method combined with a radioactivity detector and a liquid chromatography/ion trap/time-of-flight mass spectrometry. After a single oral administration, more than 94% of the dose was recovered within 14 d in the four species. The urine excretion was dominant in swine and rats, making up 78% of the dose. N-monodesmethyl-ADP, N-didesmethyl-ADP, and 10 new metabolites were characterized. These metabolites were biotransformed from the process of demethylation, α-hydroxylation, N-oxidation, and NH_2_-glucuronidation. After an oral dose for 7 d, ADP-derived radioactivity was widely distributed in tissues, and high concentrations were especially observed in bile, liver, kidney, lung, and spleen. The radioactivity in the liver was eliminated much more slowly than in other tissues, with a half-life of 4.26, 3.38, 6.69, and 5.21 d in swine, broilers, carp, and rats, respectively. ADP, N-monodesmethyl-ADP, and N-didesmethyl-ADP were the major metabolites in edible tissues. Notably, ADP was detected with the highest concentration and the longest duration in these tissues. These findings indicated that ADP is the marker residue and the liver is the residue target tissue.

2,4-Diaminopyrimidines are a class of synthetic inhibitors of bacterial dihydrofolate reductases (DHFR) acting through a blocking synthesis of DNA, RNA and proteins^1^. Aditoprim (ADP, 2, 4-Diamino-5-[4-(dimethylamino)-3,5-dimethoxybenzyl] pyrimidine) is a newly developed selective inhibitor of bacterial DHFR and can be used as an antibacterial agent in veterinary medicine. ADP exhibits excellent antibacterial efficacy *in vitro* against Gram-positive and Gram-negative bacteria. It is reported that this compound is particularly active against *Enterobacteriaceae*, *Haemophilus*, *Staphylococcus*, *Streptococcus*, *Vibrio*, and *Aeromonas*^2^. Moreover, ADP provides significant pharmacokinetic advantages, including long half-lives (6.1–11.9 h) and high tissue distribution volume (4.4–12.2 L/kg) in various animal species such as sheep^3^,^4^,^5^, cattle^6^,^7^,^8^,^9^, pig^10^, horse^11^, turkey^12^, dog^13^, and monkey^14^. These earlier studies have shown that ADP had a good efficacy when administered alone or combined with sulfonamides in a one-day dose for the treatment of respiratory and gastro-intestinal infections. Drug metabolism may generate metabolites with very different pharmaco-toxicological properties with the parent compound. In addition, the disposition processes of the parent drug and the metabolites affect the cumulative toxicity. For the food-producing animals, these processes eventually define the residue profile in edible tissues^15^,^16^. Therefore, it is vital to understand the metabolism and disposition of ADP in order to assess its potential hazard for food safety.

A few former studies have been conducted to study the *in vitro* metabolism of ADP. Mengelers *et al.* (1997) reported that ADP could be transformed into two N-demethylation metabolites, specifically, N-monodesmethyl-ADP and N-didesmethyl-ADP, upon the incubation with porcine primary hepatocytes. In addition, three unknown ADP metabolites were also observed in the chromatogram^17^. In liver microsomes from rat, cow, rabbit, sheep, pig, dog, and horse, ADP and its two N-demethylation derivatives were determined by a high-performance liquid chromatography (HPLC) method. N-monodesmethyl-ADP and N-didesmethyl-ADP were formed in all samples, but N-didesmethyl-ADP was the primary metabolite^18^. However, these former studies have only investigated the *in vitro* metabolism of ADP and just a few metabolites were identified due to the limitation of the earlier analytical methods. The *in vitro* drug metabolism study does not often fully reflect its related kinetic and dynamic processes. Up to date, the *in vivo* metabolic profiles, distribution and elimination characteristics of ADP in both laboratory animals and food producing animals still remain unknown. Moreover, with regards to ADP, no residue target tissue and marker residue have been proposed for the evaluation of food safety so far.

Isotopic tracing combined with online radioactivity detection and mass spectrometry is an effective modern analytical tool used to elucidate the metabolic and residue kinetics of the tested compounds in animals^19^. With its high resolution and mass accuracy, as well as reliable MS^n^ capability, liquid chromatography/ion trap/time-of-flight mass spectrometry (LC/MS-IT-TOF) technology is being widely used for the identification of the structure of the unknown compounds^20^. In the present study, a stable ^3^H-ADP was prepared by our group, and its metabolism, excretion, distribution, and elimination were systematically investigated in swine, broilers, carp, and rats after oral administration of ^3^H-ADP (5 mg/kg b. w.) for a single or 7 days. The radio tracer method coupled with the offline and online radioactivity detection and LC/MS-IT-TOF system were introduced in order to identify the potential metabolites in the excreta and circulation of animals, and thus will provide a quantitative assessment on the excretion routes, mass balance, tissue distribution and elimination. This study attempts to provide a comprehensive explanation of the metabolism and disposition of ADP in the species swine, broilers, carp and rats. Furthermore, the authors of this research study aim to contribute to the evaluation of ADP for the food safety.

## Results

### Radioactivity excretion

**Figure 1:**
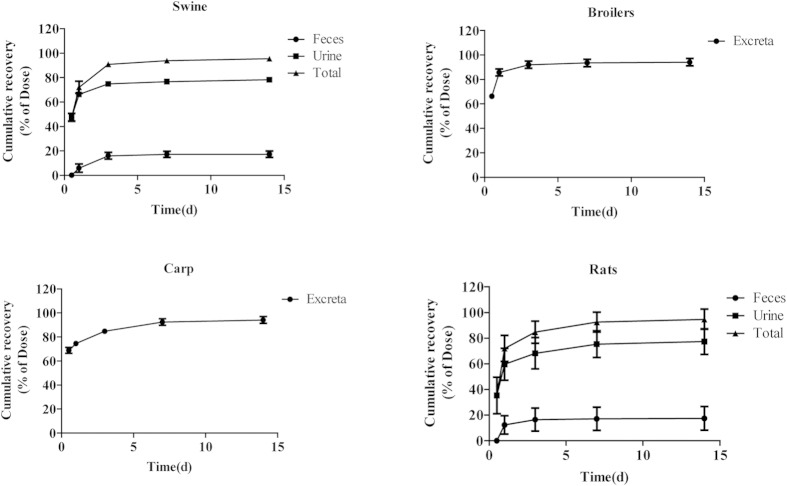
The mean cumulative excretion of radioactivity in the excreta of swine (n = 4), broilers (n = 6), carp (n = 6), and rats (n = 6) after a single oral administration of 5 mg/kg b. w ^3^ H-ADP for 14 d. The excreta were collected in the period of 6, 12, and 24 h on day 1, and afterwards, intermittently in the period of 24 h up to 14 d after dosing. The samples from the individual animals were pooled from 0 to 6 h, 6 h to 1 d, 1 to 3 d, 3 to 7d, and 7 to 14 d post-dose, respectively. The cumulative recovery was expressed as percentage of the total oral dose. During the 14-day collection period, a total of more than 94% of the radioactive dose were recovered in the four species. The peak excretion occurred during 0–6 h, followed by a slow elimination. ADP-derived radioactivity was eliminated rapidly from broilers and relatively slowly from carp. In swine and rats, the urine excretion was the primary route for elimination, making up approximately 78% of the dose. The feces were the secondary route of excretion which cleared approximately 18% of the dose.

The mean cumulative radioactivity recovery of ^3^H-ADP in the excreta of swine, broilers, carp, and rats after the single oral administration of 5 mg/kg b. w. is illustrated in Fig. 1. After the administration, the maximal excretion occurred in the period of 0–6 h and was characterized by the recovery of 47.6 ± 3.1%, 66.2 ± 1.9%, 68.8 ± 2.5%, and 35.4 ± 14.3% of the dose in the excreta of swine, broilers, carp, and rats, respectively. During one day, the values rose to 72.1 ± 4.9%, 85.8 ± 2.8%, 74.5 ± 1.7%, and 72.0 ± 10.1%, respectively. Within three days, they increased to 90.9 ± 2.2%, 92.1 ± 3.0%, 84.8 ± 2.1%, and 84.7 ± 8.6% of the dose for these four species. During a 14 d collection period, the total radioactive recovery in excreta was more than 94% of the administered dose. For swine and rats, the urine excretion was the predominant route of elimination and made up 78.3 ± 1.9% and 77.3 ± 10.0% of the administered dose within 14 d, respectively; whereas only 17.3 ± 2.5% and 17.4 ± 9.2% of the dose were recovered in feces.

### Metabolite identification

**Figure 2:**
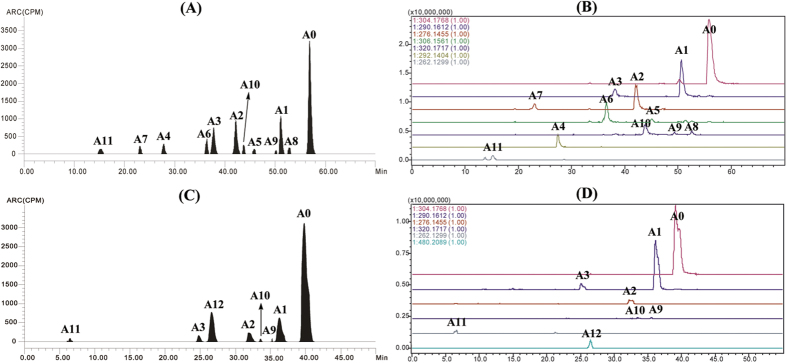
The representative HPLC radio-chromatogram (RAC) and the corresponding accurate extracted ion chromatogram (EIC) from the porcine urine (**A**) RAC, (**B**) EIC and gallinaceous feces (**C**) RAC, (**D**) EIC at 0–6 h after a single oral administration of ^3^ H-ADP (5 mg/kg b. w).

The metabolites in the excreta, plasma, and bile samples of swine, broilers, carp, and rats were qualitatively identified by the LC-*v.* ARC/MS-IT-TOF system. The metabolites were structurally characterized by comparing the changes in molecular mass, accurate mass measurements, and chromatographic retention times with those of the parent drug. Two of the metabolite references, N-monodesmethyl-ADP and N-didesmethyl-ADP were synthesized in order to confirm their structures. In the present study, a total of twelve metabolites (designated as A1 to A12) as well as parent ADP (A0) were characterized in swine, broilers, carp, and rats. A representative HPLC radio-chromatogram and corresponding accurate extracted ion chromatogram from the porcine urine and gallinaceous feces during 0–6 h is shown in Fig. 2. The [M + H]^+^ ion was generally observed in all of the metabolites. The chemical structures and supporting spectral data of ADP and its metabolites are listed in Table 1 (The accurate MS spectra of ADP and its metabolites is shown in Supplementary Information, Fig. S1).

A0 was eluted at 56.2 min and showed a protonated molecule ion at m/z 304.1746 and fragment ions at m/z 289.1597, 274.1369, 258.1337, and 123.0702. These results were compared with those of the reference substance. A0 was identified as ADP.

A1 (*t*_R_ = 51.1 min) presented a protonated molecular ion signal at m/z 290.1601, which was 14 Da lower than that of A0 and indicative of demethylation product of ADP. The fragmentation ion at m/z 275.1432 resulted from the loss of methyl, and the subsequent loss of methoxyl formed the ion at m/z 243.0864. The direct cleavage of methoxyl from the parent ion resulted in the fragment ion at m/z 260.1138. The ion at m/z 123.0782 was a characteristic fragment corresponding to the substructure of the benzyl pyrimidine ring. Therefore, this metabolite was identified as N-monodesmethyl-ADP. The structure of A1 was confirmed by comparing the retention time and mass spectrum with those of the reference substance.

A2 (*t*_R_ = 42.5 min) presented a protonated molecular ion signal at m/z 276.1452, which was 28 Da lower than that of A0, indicating that A2 is a disdemethylation product of ADP. The fragmentation ion at m/z 275.1432 resulted from the loss of methyl, following the loss of another methyl and methoxyl formed the ions at m/z 246.1069 and 232.1364, respectively. The loss of one or two methoxyl led to the formation of ions at m/z 245.1024 and m/z 216.1005, suggesting two methoxyls exist in the molecule. The ion at m/z 123.0782 was a characteristic fragment of the benzyl pyrimidine ring. Therefore, this metabolite was identified as N-disdesmethyl-ADP. The structure of A2 was also confirmed by the comparison of the retention time and mass spectrum with those of the reference substance.

A3 (*t*_R_ = 38.2 min) demonstrated the same molecular ion signal of A1, indicating that this metabolite was also a mono demethylation product of ADP. Compared with those of A1, the MS/MS fragment at m/z 274.1362 was formed from the loss of hydroxyl and indicated that demethylation occurred in the methoxyl site. The fragment ions at m/z 260.1226 and 246.1397 may be produced from the loss of methoxyl and dimethylamino, respectively. Therefore, A3 was proposed as 3-OH-ADP.

A4 (*t*_R_ = 27.3 min) had a protonated molecular ion signal at m/z 292.1416, which was 16 Da higher than that of A2, indicating that it was a hydrogenated product of disdesmethyl-ADP. The fragment ion at m/z 274.1281 was observed in the product ion spectrum, which resulted from the neutral loss of water in the ionization source, suggesting a structural modification of hydroxylation occurred in the α-H position. From the ion at m/z 274.1281, the loss of the methyl resulted in the ion at m/z 259.1070. Subsequently, a hydroxyl and methoxyl lost and produced the ions at m/z 242.0804 and 228.0912, respectively, indicating that the second demethylation was located in the dimethylamino group. Thus, A4 was tentatively identified as 3-OH-4-methylamino-ADP.

A5 and A6 were eluted at 45.1 and 36.8 min, respectively. The two metabolites exhibited the exact accurate protonated molecular ion signal at m/z 306.15, which was 16 Da higher than those of A1 and A3, indicating that A5 and A6 were hydroxylated products of A1 or A3. The fragment ion at m/z 288.1490 corresponded to the neutral loss of water from the molecular ion, indicating that this hydroxylated modification involved α-H position. Further loss of the methyl fragment generated the ion at m/z 273.1266. Compared with A5, A6 had a fragment ion at m/z 256.1221 that corresponded to the loss of hydroxyl from the ion at m/z 273.1266. Therefore, based on the fragmentation pattern and the chromatographic properties, A5 was tentatively identified as 4-methylamino-α-OH-ADP, whereas A6 was identified as 3-OH-α-OH-ADP.

A7 (*t*_R_ = 23.0 min) demonstrated a protonated molecular ion signal at m/z 276.1455, which was 28 Da lower than that of ADP, indicating that two methyls disappeared from the molecule. The fragment at m/z 261.1291 was formed by the loss of methyl from the molecular ion, and a further loss of hydroxyl resulted in the fragment ion at m/z 244.1008, indicating a hydroxyl group was located in the benzene ring. Subsequently, the loss of methyl and methylamino yielded the fragment ions at m/z 229.0879 and 216.0991, respectively. Therefore, A7 was identified as 4-methylamino-3-OH-ADP.

A8, A9, and A10 had the exact accurate protonated ion signal at m/z 320.17, which was 16 Da higher than that of ADP, which implied that these metabolites were oxydates of ADP. A8 (*t*_R_ = 52.7 min) had a fragment at m/z 288.1566 and was 32 Da lower than the parent ion produced from the loss of H_2_O and -CH_2_, indicating that hydroxylation occurred in α-H site. The fragment ion at m/z 273.1266 was generated from the loss of another methyl. Therefore, A8 was tentatively identified as α-OH-ADP. A9 and A10 had the same MS/MS fragment ions at m/z 303.1739 and 288.1470 from the loss of oxygen and methyl of the molecular ion but had different retention times (*t*_R_ values of 49.2 and 43.9 min, respectively). These results suggested that this oxidized modification most likely should occur on the nitrogen atom of the pyrimidine ring. However, the position of oxidation could not be defined. Thus, A9 and A10 were identified as 3′-N-O-ADP or 5′-N-O-ADP.

A11 (t_R_ = 14.9 min) had a protonated molecular ion signal at m/z 262.1284, which was 42 Da lower than that of ADP, corresponding to the loss of three methyls from the parent drug. The fragment at m/z 247.1043 could be attributed to the loss of methyl. Subsequently, two hydroxylation ions disappeared and generated m/z 230.0782 and 213.0770, respectively. The ion at m/z 123.0659 was a characteristic fragment of the benzyl pyrimidine ring. Therefore, this metabolite was identified as 3,5-dyhydroxy-4-methylamino-ADP.

A12 (*t*_R_ = 26.4 min) was a metabolite present only in the broiler. This metabolite had a protonated molecular ion signal at m/z 480.2125, which was 176 Da higher than that of ADP, implying that A12 was a glucuronidate metabolite of ADP. The produced ions at m/z 304.1747, 289.1507, 274.1216, and 123.0644 were similar to that of ADP. The direct glucuronidation on amino of the pyrimidine ring of ADP was proposed for metabolite A12. However, the exact site of glucuronide conjugation could not be identified.

### Metabolite profiling of ADP in different species

ADP metabolism in swine, broilers, carp, and rats was assessed by profiling the excreta, bile, and plasma samples. The extraction recovery of radioactivity from all samples was above 90% of the total radioactivity. The percentage of the individual metabolites compared with the total concentration of ADP-related for the period of 6 h after post dosing is described in Table 2.

#### Swine

ADP (A0) and 11 metabolites (A1–A11) were detected in the urine after the dose administration for 6 h. A0, A1, A2, and A3, the major radio-compounds, displayed 42.3%, 11.4%, 11.8%, and 9.7% of the sample radioactivity, respectively. During a one-day period, 9 radio-compounds (A0–A8) were detectable while only A0 and A1 could be observed in the period of 7–14 d. A0 was the primary radioactive component, representing approximately 90% of sample radioactivity. In the feces, the predominant compound A0, as well as its two metabolites (A1 and A2) were detected in the period of 0–6 h, 6 h–1 d and 1–3 d after dosing. However, only ADP (A0) was detected in the period of 7–14 d. Nine (A0, A1, A2, A4, A5, A6, A8, A9, and A10) and 6 (A0, A1, A2, A4, A5, and A6) radio-compounds were detected in the bile and plasma at 6 h and 1 d after dosing, respectively. Most of these components in the plasma and bile declined rapidly over time and only A0 was found at 7 d and after that, no metabolites were detected.

#### Broilers

A glucuronide metabolite named A12 was discovered. In the period of 0–6 h, A0, A1, and A12 were the predominant metabolites in the excreta of the broilers which were administered in a single dose, and displayed 63.2%, 12.8%, and 14.0% of the sample radioactivity, respectively. Five minor metabolites, A2, A3, A9, A10, and A11, displayed 0.2% to 6.5% of the sample radioactivity. In the period of 6 h–1 d and 1–3 d, the amount of metabolites A9, A10, A11, and A12 gradually reduced and was lower than the quantifiable level. A0 and A1 were still detectable during 3–7 d and A0 was the only one to be detectable up to 7–14 d. A0, A1, A2, and A12 were also observed in the bile and plasma after dosing for 6 h, where 51.6%, 23.4%, 12.6%, 12.4% of the sample radioactivity were detected in the bile. Around 58.4%, 28.3%, 5.8%, 4.6% (A0, A1, A2, and A12) of the sample radioactivity were found in the plasma for the period of 6 h after dosing, respectively. A4, A5, and A6 were not detected in the excreta, but were observed in the bile, and each compound displayed <2% of the sample radioactivity.

#### Carp

In the period of 6 h, the radio-compounds detected in excreta were consistent with those observed in the bile and plasma. A0, A1, and A2 displayed 87.5%, 9.9%, 2.5% of the excreta radioactivity, 64.7%, 25.5%, 9.8% of the bile radioactivity, and 61.7%, 23.9%, 14.4% of the plasma radioactivity, respectively. However, A1 and A2 were not detectable within 3 d or 7d. A0 as the most abundant component was still detectable in the excreta and bile in the period of 21 d as well as in the plasma in the period 7 d.

#### Rats

**Figure 3:**
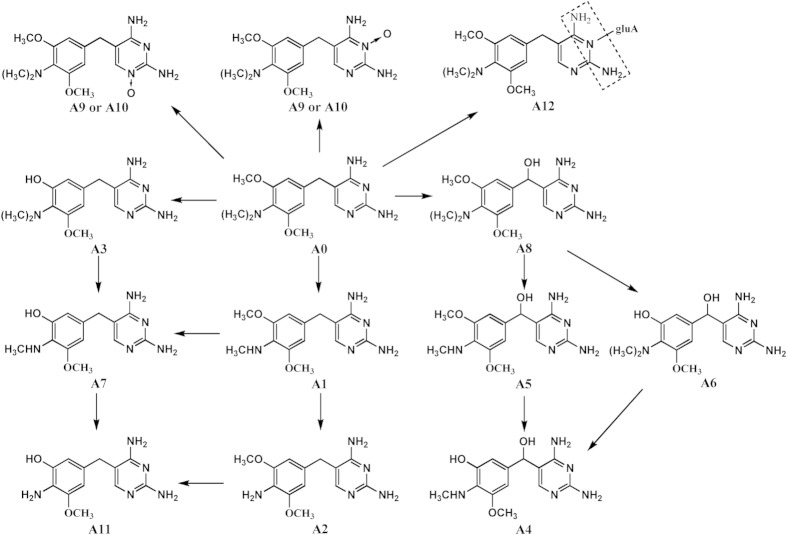
Proposed metabolic pathways of aditoprim (ADP) in swine, broilers, carp and rats.

A0, A1, A2, A6, A9, and A10 were detected in urine of the dosed rats within 6 h, representing 71.2%, 14.7%, 2.6%, 3.1%, 4.4%, and 4.1% of the sample radioactivity, respectively. These compounds, apart from A6, were still detected within 1 d. By 7 d, A0 and A1 were detected, while only A0 was detected at 14 d. A0, A1, and A2 were also detected from the feces and plasma at 6 h, where they exhibited 80.9%, 14.8%, 4.3% of the feces radioactivity and 75.0%, 19.0%, 6.0% of the plasma radioactivity, respectively. Based on the putative structure, content variation, and persistence of metabolites, a proposed metabolic pathway for ADP in the four species is presented in Fig. 3.

### Tissue distribution and elimination of the total radioactivity and individual radio-compounds

**Figure 4:**
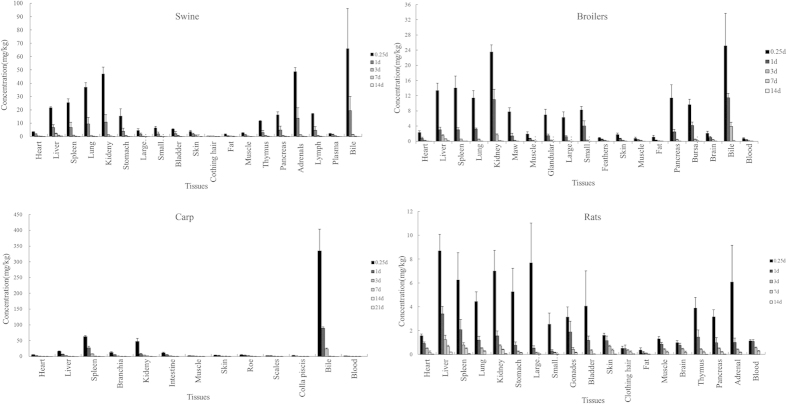
The tissue distribution for ADP in swine, broilers, carp, and rats after a multi-dose of ^3^H-ADP at 5 mg/kg b. w. for 7 d. The animals were slaughtered in the period of 6 h and 1, 3, 7, 14, and 21 d after the administration. The values are expressed as averages ± S.D. Similar trends of the distribution profile were displayed among all the four species. The drug-related radioactivity underwent a rapid and wide distribution, and reached the highest concentration in various tissues and fluids during 6 h after dosing. The high levels of concentrations were observed in the metabolic/excretory (bile, liver, and kidney), respiratory (lung), and endocrine (spleen, thymus gland, pancreatic, lymph, and adrenal) systems. The drug related radioactivity rapidly declined in the period of 24 h after the administration. Afterwards, it decreased slowly, and was still detected in almost all tissues in the period of 7 d after dosing. By day 14 (21 d for carp), the radioactivity could still be detected in the heart, liver, spleen, and kidney in the four species.

Following an oral administration of ^3^ H-ADP for 7 d, the tissue distribution profiles of the total radioactivity were similar among these four species (Fig. 4). The drug-related radioactivity underwent a rapid and wide distribution, and reached the highest concentration in various tissues and fluids within 6 h in all four species. The highest concentrations were found in the bile (66.0 ± 30.1, 25.2 ± 8.5, and 334.6 ± 69.0 mg/kg for swine, broilers, and carp, respectively); followed by the kidney, liver, lung, carp gills, spleen, pancreas, adrenal gland, thymus, and bursa of fabricius, with concentrations ranging from 10 to 50 mg/kg. A low level of radioactivity was observed in the gastrointestinal tract, heart, skin, carp scales, muscle, fat, brain, hair, carp maw, and blood, with concentrations ranging from 1 to 10 mg/kg. The concentrations of radioactivity in the tissues were generally higher than those in the blood at almost all time points. The drug related radioactivity rapidly declined during 24 h after the administration. After that, it decreased slowly and was still detectable in almost all tissues within 7d after dosing among these four species. By 14 d (21 d for carp), after the dose administration, the radioactivity could still be detected in the heart, liver, spleen, and kidney. The elimination rates for radioactivity were estimated in all tissues by a linear regression analysis of concentrations in the terminal phase (Supplementary Information, Table S1–4). Generally, in comparison with the slowest depletion in carp with *t*_1/2_ ranging 1.83–6.69 d, broilers showed a faster depletion rate with *t*_1/2_ ranging 0.77–3.38, followed by swine and rats with *t*_1/2_ ranging 0.97–4.26d, and 1.23–3.96 d, respectively. The longest residue time of the total radioactivity was observed in the liver and then in the kidney for swine, broilers, carp, and rats, with *t*_1/2_ of 4.26, 3.38, 6.69, and 3.96 d for the liver, and 3.35, 2.33, 3.56, and 3.23 d for the kidney, respectively.

The concentrations of ADP and its metabolites in the liver, kidney, muscle, and fat (skin for carp) of swine, broilers, carp, and rats are shown in Tables 3 and 4. A total of 12 (A0–A11), 8 (A0, A1, A2, A3, A9, A10, A11, and A12), 3 (A0, A1, and A2), and 6 (A0, A1, A2, A6, A9, and A10) radio-compounds were detected in the major tissues of swine, broilers, carp, and rats, respectively. Most of the compounds were observed in the liver and kidney, but only three compounds (A0, A1, and A2) were observed in the muscle and fat (skin for carp). In swine, A0–A11 were detected in the liver at 6 h after the dose administration. These compounds, except A3, A7, and A8, were also detected in the kidney. In broilers, A0, A1, A2, A9, A10, and A12 were the common metabolites in the liver and kidney, but A11 and A3 was only detected in liver and kidney, respectively. In rats, A0, A1, A2, A9, and A10 were the common metabolites in the liver and kidney. In addition, A6 was detected in rat liver. In carp, only A0, A1, and A2 were detected in the liver and kidney. A0, A1, and A2, the major radioactive components in the four species, could be consistently detected within 3 d after the last dosing. Other minor metabolites depleted rapidly and vanished within 1 d or 3 d. A0 was the only detectable compound after the dose administration for 7 d or 14d. ADP was the most predominant component in all detected tissues among these four species, represented more than 42% of the sample radioactivity at all the sampling times. A1 as one of the major metabolites of ADP, exhibited 18.2–34.1%, 21.3–26.8%, 12.4–16.8%, 19.2–38.5% of the sample radioactivity in swine, broilers, carp, and rats, respectively. Another major metabolite A2, displayed 4.0–14.1%, 1.6–7.9%, 1.6–2.9%, 3.1–5.3% of the sample radioactivity in the four species, respectively. The kinetic parameters of these major compounds are illustrated in Table 5. Similar to the elimination tendency of the total radioactivity, individual compounds were eliminated more rapidly in broilers, but relatively more slowly in carp. ADP was the slowest eliminated component with *t*_1/2_ ranging 1.62–4.38, 1.67–3.40, 2.40–6.63, and 2.59–4.17 d in swine, broilers, carp, and rats tissues, respectively, followed by A1 with *t*_1/2_ ranging 0.73–1.04, 0.68–1.05, 1.09–2.81, and 2.11–2.69 d in the tissues of all four species. A2 was eliminated quickly, with *t*_1/2_ ranging from 0.42 to 1.61 d. Moreover, *t*_1/2_ could be only calculated in the kidney and liver of swine and rat.

## Discussion

This radio-labeled study evaluated the metabolism and disposition fate of ADP and explained its route of excretion and metabolism with the purpose to eliminate the radioactivity in swine, broilers, carp and rats. The cumulated recovery in the four species was above 94% after the administration during the 14-day collecting period, suggesting that no significant accumulation of ADP and its metabolites in the tissues occurred. The urine excretion was the major excretion route for ADP, making up approximately 78% of the dose in swine and rats. This observation indicated that ADP had a good absorption profile in these species. The excretion pathway was similar to its analogs TMP and metioprim, with 66–85% of the dose recovered in the urine after the oral administration^21^,^22^. In contrast to the excretion of TMP in rats, more than 95% of the oral dose was recovered within 3 d. ADP showed a relatively slow elimination trend (approximately 84% of the oral dose during 0–3 d) in comparison with the rat excreta, implying that ADP would have a longer elimination half-life than TMP in the organism. This finding was further supported by the pharmacokinetic characteristics of ADP in animals^5^,^6^,^8^. ADP related radioactivity in broilers was excreted more rapidly than in other species, with a high recovery of 85.8% (about 70% in other species, Fig. 1) of the dose within 1 d. This difference might be caused by the shorter gastrointestinal tract in broilers^23^. A slight discrepancy of excretion rate was also observed among the other examined species. Body size, energy turnover, and basal metabolic rate also influence drug elimination according to allometric relationships^24^.

The metabolic profile analysis indicated that ADP underwent extensive metabolic reactions among the four species. We found that ADP could be metabolized more extensively in swine than in the other tested species. Nearly all the metabolites detected in broilers, carp, and rats were observed in swine, except for A12, which was only detected in broilers. Carp had a comparatively weak metabolic ability for ADP, since only two metabolites were detected in this species. Based on our study, the metabolic pathways of ADP are involved in N-demethylation, O-demethylation, α-hydroxylation, N-oxidation, and additional glucuronide conjugation. N-demethylation as the major metabolic pathway remained consistent in all four species, leading to the generation of two major metabolites, A1 and A2. Our result was in accordance with the earlier *in vitro* metabolic profiles of ADP in hepatocytes or microsomes from various animal species, where two N-demethylation derivatives were detected^17^,^18^. We propose in the light of the these former studies that the liver is the main metabolic organ of ADP. In contrast to TMP, diaveridine, and brodimoprim, O-demethylation were the major metabolic pathways in laboratory rats, target species, and humans^21^,^25^,^26^,^27^,^28^,^29^. If the phenyl moiety is important for the affinity of the drug to the O-demethylating enzyme, then the presence of a dimethylamino group may induce steric hindrance for this enzyme, resulting in the markedly different profiles in the major metabolic pathway. Some minor metabolic pathways of ADP, including O-demethylation, α-hydroxylation, N-oxidation, and NH_2_-glucuronidation were detected for the first time *in vivo*. They also played an important role in the compound clearance, because 10 metabolites (A3–A12) were involved in these pathways. Conjugated derivatives were detected only in broilers. In broilers, the glucuronide product A12 was detected in the excreta, plasma, and bile with a relatively high percentage of 4.6–14.0% in the period of 6 h after dosing, indicating that the species-related differences in the biotransformation enzyme expression of ADP were present^30^. On the other hand, this observation could be associated with the relatively rapid elimination of ADP in broilers, which was caused by stronger hydrophilicity of the conjugate derivatives^31^. In carp, the level of intact ADP was significantly higher (82.4% of the oral dose) than that in other species (49–75% of the oral dose) and only 2 metabolites (A1 and A2) were detected (Supplementary Information Table S5). The metabolic stability of ADP might contribute to the long half-life of ADP in carp^32^.

The studies of the tissue distribution indicated that the ADP-related radioactivity is widely distributed. The peak concentration in all tissues was observed within 6 h after the dose administration. The highest level of radioactivity was observed in the bile, whereas the higher radioactivity recovery in the urine (78% of the administered dose compared to 18%) indicated a biliary excretion and a subsequent reabsorption. The concentrations of the radioactivity in the kidney, liver, lung, spleen, and thymus/bursa were significantly higher than in the other tissues. The adequate concentration of ADP in the lung seems to contribute to its excellent antibacterial effects on the prevention and treatment of pulmonary infections caused by sensitive bacteria *Pasteurella multocida* and *Actinobacillus pleuropneumoniae*^33^. However, high radioactivity levels in those tissues indicated that they would be easily exposed to the drug and/or its metabolites in toxicity studies^34^, which resulted in obvious organ injure when dosed at higher level^35^. Within 14–21 days after dosing, the high radioactivity levels were still detectable in the kidney and liver, suggesting that the kidney and liver should be the key tissues for food safety control and toxicology concern. The comparison of half-times clearly showed that the radioactivity elimination from the liver was much slower than that of the kidney and other tissues. Those results revealed that the liver is the appropriate target tissue for the residue monitoring. In the depletion study of TMP, TMP as a marker residue could be detected at early time points and below the LOD by 7 d^36^. In the present study, ADP as the predominant component in all samples was detectable within 14–21 d of administration. Similar with TMP, the side chain of dimethylamino group on ADP lies outside of the hydrophobic cleft of the DHFR and thus has a minimal contact with the enzyme^37^. However, the increases in the lipophilicity and tissue penetration may result in a higher tissue distribution and longer half-life than TMP^38^. Based on the similar concentration–time curve and elimination half-life in residue target tissue (Supplementary Information, Fig. S2), ADP is proposed as the marker residue in swine, broiler, and carp.

The use of trimethoprim (*t*_1/2_ = 2.5–3 h)/sulfonamide (*t*_1/2_ > 8 h) combinations is controversial because the ideal concentration ratio of the two drugs for optimum synergism has not been established for the TMP absence in the tissues for a large proportion of the inter-dose interval^39^,^40^. The superior properties of ADP associated with minimal biotransformation allow its administration only once a day. In addition, the N-desmethyl metabolites did not modify the basic pteridine structure, which would be expected to exhibit some microbiological activities^36^. Therefore, ADP would exhibit even a stronger antimicrobial efficacy *in vivo* and has a potential to be a single entity drug for the inhibition of bacterial folic acid synthesis, although its combination with an appropriate sulfonamide should be considered.

## Materials and Methods

### Chemicals

Tritium-labeled ADP was synthesized at the Chinese Academy of Science Shanghai Institute of Applied Physics (Shanghai, China). Pd/C (10%) was purchased from Sigma-Aldrich (Milwaukee, WI, USA). The reference substances of ADP and metabolites A1, A2 with chemical purity > 98% were synthesized at the National Reference Laboratory of Veterinary Drug Residues (Wuhan, China). ULTIMA GOLD scintillation fluid and Solvable^TM^ digestion solution were purchased from Perkin–Elmer Inc. (Massachusetts, USA). ^3^ H cocktail for online *v.* ARC radioactive detection was purchased from XenoBiotic Laboratories, Inc. (Nanjing, China). HPLC-grade methanol was obtained from Fisher Chemicals Co. (New Jersey, USA). Water was purified by using a Milli-Q system (Bedford, MA, USA). All other chemicals and reagents are analytical grade or higher purity.

#### Tritium-labeled ADP

The synthesis procedure for ^3^H-ADP is illustrated in Supplementary Information, Fig. S3. Compound **1** (3.0 g, 0.015 mol) was dissolved in 100 mL of methanol, and the solution was added with 0.68 g of NaBH_4_ (0.02 mol). The reaction mixture was stirred at room temperature for 1 h to produce compound **2**. Subsequently, compound **2** (2.12 g, 0.01 mol) was dissolved in dichloromethane (100 mL), 40% HBr (2.02 g, 0.01 mol) and 30% H_2_O_2_ (0.39 g, 0.015 mol) were added. The obtained solution was stirred at 5–10 °C for 5 h to yield compound **3**, which was purified through column chromatography on silica gel with dichloromethane–methanol (1:20, v:v) as eluent. Compound **3** (2.90 g, 0.01 mol) was reacted with oxalyl chloride (1.43 mL, 0.015 mol) and dimethylsulfoxide (1.42 mL, 0.02 mol) in dry dichloromethane under Swern oxidation conditions^41^ to obtain compound **4**. The knoevenagel reaction was performed with compound **4** (3.54 g, 0.01 mol) and 3-methoxypropionitrole (1.90 mL, 0.02 mol) in methanol solution of sodium methoxide at 30 °C for 2 h and produced compound **5**. Under the treatment of sodium ethylate, compound **5** (2.00 g, 0.005 mol) had a cyclization effect with guanidine carbonate (1.80 g, 0.01 mol) at reflux temperature for 5 h and compound **6** was obtained and recrystallized from methanol. Compound **6** (60.0 mg, 0.175 mol) was dissolved in 3 mL of methanol–DMF solution (1:1, v:v) and added with 25 mg of 10% Pd/C. The mixture was reacted with 250 mmHg tritium gas (T_2_) at 60 °C for 2 h. The reaction mixture was filtered after the recycle of the excess T_2_, and was concentrated to obtain crude 2−^3^ H-ADP. Subsequently, they were purified in the model 2695 HPLC with a 2996 detector (Waters, Milford, MA). A Venusil XBP-18 column (10 μm, 150 mm × 21.5 mm; Agela Techonologies, Inc., China) was used for separation of the mixture. The mixture was eluted with methanol/0.1% ammonia water (1:1, v:v) at a flow rate of 5 mL/min at 30 °C. The fractions containing the pure ^3^ H-ADP were pooled, concentrated, lyophilized, and dissolved in absolute ethyl alcohol to yield 2−^3^ H-ADP, with a specific activity of 10 mCi/mol, chemical purity of 99.0%, and radiochemical purity of 99.2%. The obtained solution was stably when stored at −20 °C for 6 months, and the tritium exchange risk in all samples was confirmed to be <5%.

### Animals

Swine (60 day old, 30 ± 2 kg) were supplied by China Breeding Swine Testing Center (Wuhan, China). Chicken broilers (40 day old, 2.0 ± 0.1 kg) were purchased from Wuhan Chia Tai chicken farm (Wuhan, China). Carp (0.5 ± 0.05 kg) were supplied by the Aquatic Product Base in Huazhong Agricultural University (Wuhan, China). Specific pathogen-free Wistar rats (8 week old, 200 ± 20 g) were obtained from Hubei Experimental Animal Research Center (Wuhan, China). The animals were maintained under the standard environmental conditions by using normal husbandry practices and allowed to acclimatize for a week prior to the study. The room temperature and humidity was maintained at 20–25 °C and 50–60%, respectively. The animals were housed in a 12 h light/dark cycle. Carp were held in the identical, 1.22-m-diameter circular fiberglass aquaria, which was filled with 50 L naturally aerated water (25 ± 3 °C). The water was continuously received flowing fresh air from the same Aero-tube^TM^ aeration system. All the animals were fed with their standard feeds *ad libitum.* All experimental procedures were conducted in accordance with the guidelines of the VICH Guideline GL.46 (2012)^42^ and the FDA Good Laboratory Practice for Nonclinical Laboratory Studies^43^.

All animal care and experimental protocols were conducted in accordance with the Guide for the Care and Use of Laboratory Animals of Hubei Provincial Laboratory Animal Public Service Center (permit number SYXK 2013-0044) and approved by the Ethics Committee of Huazhong Agricultural University, Wuhan, China.

### Dose preparation and sample collection

The oral dose formulation was prepared by accurately mixed amounts of ^3^ H-ADP and non-labeled ADP in a vehicle of 0.5% methylcellulose with a specific activity of 0.1 Ci/g. Aliquots of the solution were analyzed by using a liquid scintillation counter (LSC, Tri-carb 2900TR, Perkin–Elmer, Inc.) to confirm the dose homogeneity. Radioactivity and radiochemical purity of ^3^ H-ADP were determined before the preparation of each dose formula. The individual animal doses were calculated on the basis of respective pretreatment body weight (5 mg/kg b. w.) and the dose volume appropriate for the specific species. The actual content of the administered dose solution was checked by weighing the loaded dosing syringe before the administration and the emptied syringe after the administration.

#### Excretion and metabolism of ^3^ H-ADP

Animals (4 swine, 6 broilers, 6 carp, and 3 male and 3 female rats) were housed individually in clean, suspended wire-mesh metabolism cages, designed to separate the urine from the feces (carp were raised in individual aquaria). Each animal was weighed and assigned with a permanent identification number. A single dose of ^3^ H-ADP (5 mg/kg b. w., 0.1 Ci/g) was administered via the oral gavage. Excreta were collected on ice at the pre-administration, in the period of 6, 12, and 24 h on day 1 d, and afterwards, intermittently in the period of 24 h up to 14 d. The piscine excreta were collected by changing and collecting raised water. The total volume or weight of each sample was measured and homogenized. The total radioactivity levels were determined through LSC for the recovery calculations. After the preparation, the samples were analyzed through LC/MS-IT-TOF with *v.* ARC radiometric detection (XenoBioticLaboratories, Inc.) for metabolite profiling. The pretreatment samples were used as controls to determine the background radioactivity or the endogenous nondrug-related ions observed within respective matrices or their extracts.

#### Distribution and elimination of ^3^ H-ADP

A total of 36 animals from each species (swine *n* = 24) were randomly assigned to six equal treatment groups and administered a single daily dose of ^3^ H-ADP at 5 mg/kg b. w. (0.1 Ci/g) through oral gavage for 7 consecutive days. All the animals were housed on the basis of their group in the cages or aquaria throughout the whole study period. A group of animals was slaughtered through exsanguination under diethyl ether inhalation of anesthesia in the period of 6 h, 1, 3, 7, 14, and 21 d after the administration. The blood samples were immediately collected into the heparinized tubes and was centrifuged at 1920 × g at 4 °C for 10 min to obtain the plasma. The tissues from the heart, liver, spleen, lung, kidney, craw (broilers), stomach, intestine, fat, skin, muscle, thymus, pancreas, bile, bladder, hair (scale for carp), genital gland, adrenal glands, swim bladder (carp), brain, lymph, and bursa (broilers) were carefully excised and collected. The tissues were thoroughly rinsed with the deionized water to remove the residuary blood, blotted to dryness, and finally to perform the homogenization. In order to avoid cross-contamination the dissecting instruments were washed during the tissue procurements. The blank tissues were collected in order to provide control matrices. The total radioactivity levels in each sample were determined through LSC. The samples extracted from the plasma, bile, and tissues of the liver, kidney, muscle, and fat/skin were analyzed through LC/MS-IT-TOF and *v.* ARC radiometric detection for metabolite profiling. The blank samples were used as control to determine the background radioactivity or the endogenous nondrug-related ions observed within respective matrices or their extracts.

### Assay of the total radioactivity

Measurements of the total radioactivity in each sample were conducted by Packard Tri-Carb 2900 TR liquid scintillation counter (PerkinElmer Life and Analytical Sciences). The detection time was 10 min for two cycles. The scintillation counter data were automatically corrected for counting efficiency by instrument-stored quench curve which was drawn with a series of sealed quenched ^14^ C standards. The actual radioactivity of each sample was calculated by subtracting the background value form the mean radioactivity in two blank samples. All the samples were analyzed in duplicate if the sample size allowed this. If the results were differed by more than 10% from the mean value, the samples were reanalyzed. The samples with LSC counts less than 2-fold of the level of the running background were considered to contain insufficient radioactivity for reliable quantification.

Aliquots of liquid samples (urine, plasma, bile and raised water, 200 μL) were mixed with 10 mL of Ultima Gold^TM^ scintillation fluid for the direct analysis by LSC. Aliquots of solid samples (feces and tissues, 200 mg) were digested overnight with 2 mL tissue solubilizer at 50 °C in a shaking water bath, followed by adding 200 μL of 0.1 mol/L EDTA-Na and 200 μL of 30% H_2_O_2_, and was bathed at 50 °C for 2 h to bleach. The obtained transparent liquid were mixed with 10 mL of scintillation fluid and finally were counted by LSC. The digestion efficiency was determined by using the blank samples spiked with ^3^ H standard for digestion, and the measured radioactivity content in the digested samples were adjusted by using the digestion efficiency values.

### Extraction of metabolites from the biological samples

For metabolite profiling, the urine and feces samples from each animals were pooled proportionally to the excreted amount in each sampling period from 0 to 6 h, 6 h to 1 d, 1 to 3 d, 3 to 7 d, and 7 to 14 d post-dose, respectively. All samples were fully homogenized before the process. The pre-dose and blank samples served as the controls for determining the background radioactivity or the endogenous, nondrug-related ions observed within respective matrices or their extracts.

The urine samples (plasma, bile and raised water for carp) was subjected to solid-phase extraction with an Oasis MCX cartridge (3 mL, 6 g; Waters, Milford, MA, USA) for metabolite purification and enrichment. The pH value was adjusted to 2.8–3.2 by adding 1 M hydrochloric acid (HCl), and 2 mL of the sample was transferred into MCX cartridge, which was preconditioned with 3 mL of methanol and 3 mL of 0.1 mol/L HCl solution. The cartridge was washed with 3 mL of 0.1 mol/L HCl solution and was eluted with 5 mL of NH_4_OH/methanol (4:96, v:v). The effluent was concentrated to dryness under a stream of nitrogen at 40 °C. The residue was reconstituted in 0.3 mL of methanol–water (1:9, v:v) before the injection into the LC-*v.* ARC/MS-IT-TOF system for the identification and quantification of the potential metabolites.

The feces and tissues (2 g) were diluted with a mixture of methanol and chloroform (1:3, v:v; 2 mL/g homogenate). This procedure was carried out in a fume hood for the safety aspects. The pH value was adjusted to 9.0 with NH_4_OH. The mixture was vortexed and sonicated for 5 min and 10 min, respectively, and finally the samples were centrifuged at 7690 × g for 10 min. The supernatant was aspirated and then transferred into a new centrifuge tube. The obtained pellet was again subjected to the same process of vortex, ultrasonication, extraction, and centrifugation. The combined supernatant was concentrated under a stream of nitrogen in a water bath at 40 °C. The residue was reconstituted with 0.3 mL of methanol–water (1:9, v:v) before the injection into the LC-*v.* ARC/MS-IT-TOF system for analysis.

### Quantification and structural identification of metabolites

ADP and its metabolites in the prepared samples were determined with a *v.* ARC radiometric detector and a hybrid IT/TOF mass spectrometer connected to the HPLC system (Shimadzu Corp., Kyoto, Japan). The LC system was equipped with a solvent delivery pump (LC-20AD), an auto-sampler (SIL-20AC), a degasser (DGU-20 A 3), a photodiode array detector (SPD-M20A), a communication base module (CBM-20 A), and a column oven (CTO-20AC). The chromatographic separation was achieved by using an Agilent extend-C18 column (2.1 mm × 150 mm, 5 μm) with a gradient mobile phase of A (0.01% ammonia and 5 mM ammonium acetate in water) and B (methanol) at a flow rate of 0.2 mL/min. The column temperature was maintained at 35 °C. Various HPLC methods were performed because of the large number of different metabolites to be analyzed. For the porcine sample, method 1 started at 5% B and increased to 20% B over 35 min, then to 55% B by 60 min, followed by re-equilibration for another 10 min. For the remaining samples, method 2 started at 10% B and increased to 60% B over 30 min, subsequently was maintained at 60% B for an additional 10 min, and then changed to 90% B over 2 min and maintained for 2 min. Afterwards, a re-equilibration step (decreased to 10% B over 1 min and maintained at 10% B for additional 9 min) was followed. For each matrix, more than 98% of the radioactivity injected into the column was eluted during the running time of the gradient program.

HPLC column effluent was split to 1:9 between the radiometric flow detector and mass spectrometer. The flow cell volume was 0.5 mL, and liquid scintillation cocktail flowed into a dynamic flow mode. The limit of quantification (LOD) for radioactivity was defined as the ratio of signal-to-noise (3:1) on the radio-chromatogram. The radioactive contents of the separated components were determined through the integration of the corresponding radioactive peaks by using the *v.* ARC software. The conversion was performed by using the following linear equation: *Y* = 7.956*X* + 171.8 (*R*^2^ = 0.9991), where *X* (cpm) was obtained from the *v.* ARC and *Y* (dpm) from Tri-Carb 2900TR.

The structural characterization was conducted by LC-*v.* ARC/MS-IT-TOF which was equipped with an electrospray ionization source in a positive ion mode. The mass spectrometric analyses were conducted by full-scan MS with a mass range of 100–800 Da and data-dependent MS^n^ acquisition of the suspected metabolite ions. Liquid nitrogen was used as nebulizing gas at a flow rate of 1.5 L/min. The capillary and skimmer voltages were set to 4.5 and 1.65 kV, respectively. The CDL and heat block temperatures were maintained at 200 °C. The MS^2^ spectra were produced by using the CID of the selected precursor ions with argon as collision gas (relative energy of 50%). The ion accumulation time were set to 30 ms, and the precursor ion isolation width at 1 Da. The external mass was calibrated prior to the data acquisition by using direct infusion of the reference standard from 50 Da to 1000 Da. The reference standard consisted of 0.25 mL/L trifluoroacetic acid and 0.1 g/L sodium hydrate at a flow rate of 5 mL/min. All calculated mass errors were smaller than 5 mDa after the mass calibration with the reference standard. The fully characterized metabolites were designated by letter A and followed by a number.

### Data analysis

The concentrations of ADP and/or its metabolites were analyzed by using the following equation: C = L/(2.22 × 10^9^ × RM), where C is the concentration of ADP or/and its metabolites (mg/kg), L is the LSC count (dpm), R is the specific activity of the administered dose (0.1 Ci/g), and M is the weight or volume of samples (kg or L). The results are expressed as mean ± SD. The elimination kinetics of terminal phase for ADP and its metabolites were conducted by using the WinNonlin linear regression program (Pharsight corporation, Statistical consultants, Inc., Kentucky, USA).

## Additional Information

**How to cite this article**: Wang, L. *et al.* Metabolism and Disposition of Aditoprim in Swine, Broilers, Carp and Rats. *Sci. Rep.* **6**, 20370; doi: 10.1038/srep20370 (2016).

## Supplementary Material

Supplementary Information

## Figures and Tables

**Table 1:**
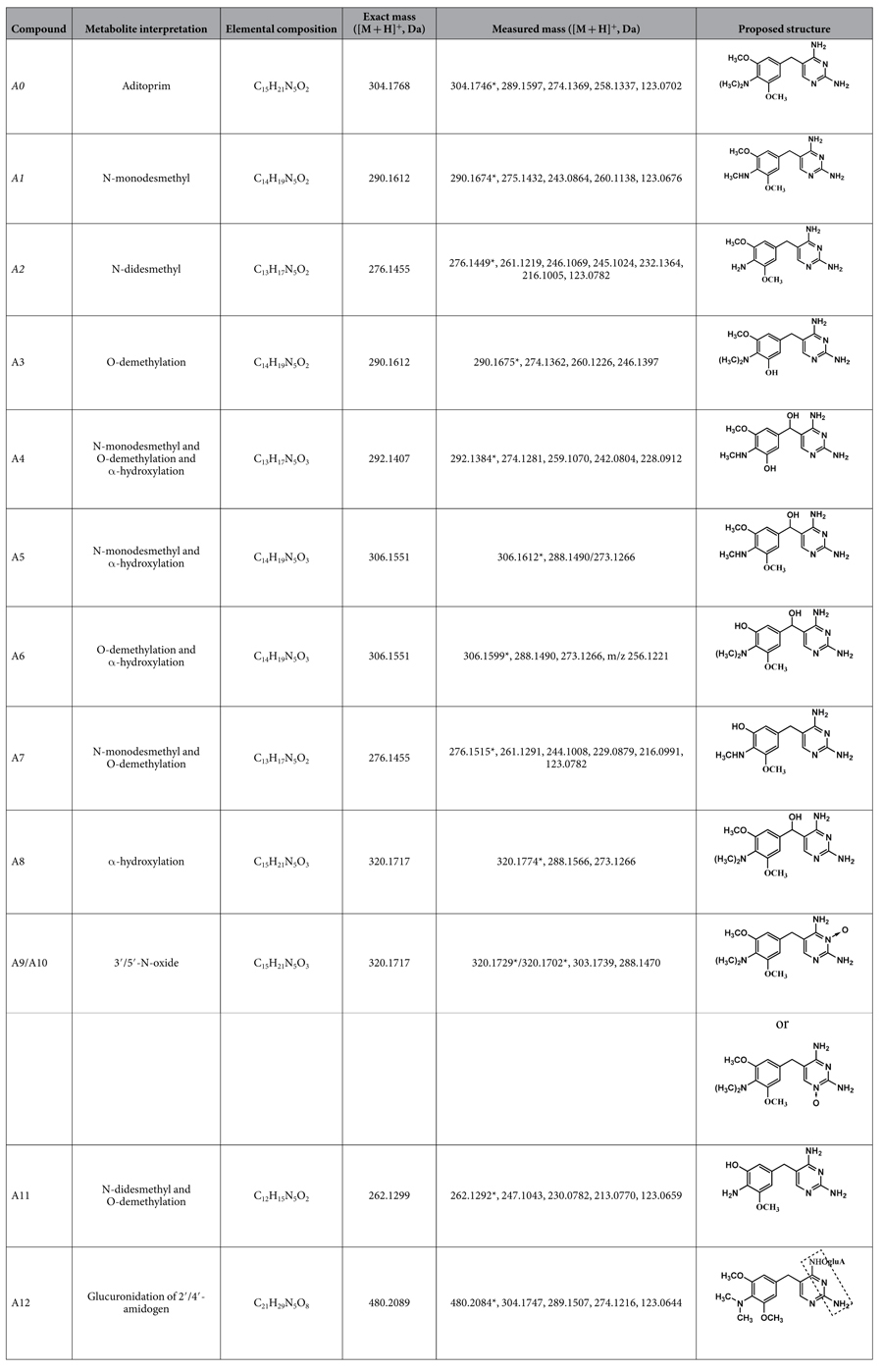
The chemical structures and supporting spectral data of ADP and its metabolites.

Italicized metabolites were confirmed with comparison with reference substance. *Represented the measured parent ion ([M + H] ^+^ ) and other represented the measured fragment ions ([M + H] ^+^ ). The measured and theoretical mass errors were < 5 mDa.

**Table 2:** The percentage of the individual metabolites compared with the total concentration of ADP-related within 6 h after dosing (%).

	Swine			Broilers				Carp			Rats		
	Urine	Feces	Plasma	Bile	Extra	Plasma	Bile	Extra	Plasma	Bile	Urine	Feces	Plasma
A0	42.3	89.1	53.0	21.8	63.2	51.6	58.4	87.5	61.7	64.7	71.2	79.4	75.0
A1	11.4	9.7	23.7	17.3	12.8	23.4	28.3	9.9	23.9	25.5	14.7	20.4	19.0
A2	11.8	1.1	15.0	12.5	6.5	12.6	5.8	2.5	14.4	9.8	2.6	0.3	6.0
A3	9.7	−	−	−	3.5	−	−	−	−	−	−	−	−
A4	4.8	−	4.6	11.0	−	−	1.2	−	−	−	−	−	−
A5	1.2	−	1.6	4.5	−	−	1.0	−	−	−	−	−	−
A6	6.4	−	3.8	28.3	−	−	1.8	−	−	−	3.1	−	−
A7	1.9	−	−	−	−	−	ND	−	−	−	−	−	−
A8	1.4	−	−	3.1	−	−	ND	−	−	−	−	−	−
A9	0.7	−	−	0.5	0.2	−	ND	−	−	−	4.4	−	−
A10	5.5	−	−	0.6	0.2	−	ND	−	−	−	4.1	−	−
A11	1.6	−	−	−	0.3	−	ND	−	−	−	−	−	−
A12	−	−	−	−	14.0	12.4	4.6	−	−	−	−	−	−

Data was not detected.

**Table 3:** Concentration of ADP and its metabolites in tissues of swine (n = 4) dosed ^3^ H-ADP for 7 days.

Tissue	Time(d)	Concentration (mg/kg)											
		A0	A1	A2	A3	A4	A5	A6	A7	A8	A9	A10	A11
Liver	0.25	7.60 ± 1.04	4.62 ± 0.85	2.44 ± 0.48	0.94 ± 0.17	0.32 ± 0.03	0.43 ± 0.04	0.36 ± 0.04	0.79 ± 0.17	0.02 ± 0.00	0.19 ± 0.02	0.32 ± 0.03	0.78 ± 0.08
	1	3.01 ± 0.90	1.65 ± 0.33	0.84 ± 0.11	ND	0.27 ± 0.04	0.26 ± 0.04	ND	0.45 ± 0.09	ND	ND	ND	0.22 ± 0.03
	3	1.32 ± 0.27	0.64 ± 0.11	0.31 ± 0.08	ND	ND	ND	ND	ND	ND	ND	ND	ND
	7	0.82 ± 0.14	ND	ND	ND	ND	ND	ND	ND	ND	ND	ND	ND
	14	0.24 ± 0.05	ND	ND	ND	ND	ND	ND	ND	ND	ND	ND	ND
Kidney	0.25	19.02 ± 8.81	15.36 ± 5.3	6.36 ± 1.04	ND	0.52 ± 0.08	0.71 ± 0.11	0.69 ± 0.10	ND	ND	0.85 ± 0.13	0.47 ± 0.07	1.25 ± 0.18
	1	4.16 ± 2.00	3.43 ± 1.25	1.30 ± 0.40	ND	0.48 ± 0.26	0.27 ± 0.15	0.06 ± 0.03	ND	ND	0.30 ± 0.16	0.02 ± 0.01	ND
	3	1.08 ± 0.22	0.16 ± 0.04	0.02 ± 0.01	ND	ND	ND	ND	ND	ND	ND	ND	ND
	7	0.36 ± 0.09	0.02 ± 0.00	ND	ND	ND	ND	ND	ND	ND	ND	ND	ND
	14	0.11 ± 0.02	ND	ND	ND	ND	ND	ND	ND	ND	ND	ND	ND
Muscle	0.25	1.49 ± 0.39	0.85 ± 0.25	0.16 ± 0.04	ND	ND	ND	ND	ND	ND	ND	ND	ND
	1	0.89 ± 0.22	0.15 ± 0.04	0.05 ± 0.01	ND	ND	ND	ND	ND	ND	ND	ND	ND
	3	0.38 ± 0.09	0.05 ± 0.01	ND	ND	ND	ND	ND	ND	ND	ND	ND	ND
	7	0.07 ± 0.01	ND	ND	ND	ND	ND	ND	ND	ND	ND	ND	ND
	14	ND	ND	ND	ND	ND	ND	ND	ND	ND	ND	ND	ND
Fat	0.25	1.27 ± 0.43	0.30 ± 0.07	0.07 ± 0.01	ND	ND	ND	ND	ND	ND	ND	ND	ND
	1	0.51 ± 0.12	0.06 ± 0.01	ND	ND	ND	ND	ND	ND	ND	ND	ND	ND
	3	0.33 ± 0.07	ND	ND	ND	ND	ND	ND	ND	ND	ND	ND	ND
	7	0.15 ± 0.03	ND	ND	ND	ND	ND	ND	ND	ND	ND	ND	ND
	14	ND	ND	ND	ND	ND	ND	ND	ND	ND	ND	ND	ND

ND: Data was not detected.

**Table 4:** Concentration of ADP and its metabolites in the tissues of broiler, carp and rat after the administration of ^3^ H-ADP for 7 day (n = 6).

Tissue	Time (d)	Concentration (mg/kg)																
		Broilers								Carp			Rats					
		A0	A1	A2	A3	A9	A10	A11	A12	A0	A1	A2	A0	A1	A2	A6	A9	A10
Liver	0.25	8.04 ± 1.40	3.09 ± 0.68	0.38 ± 0.05	ND	ND	ND	0.31 ± 0.11	0.70 ± 0.19	13.22 ± 3.70	1.93 ± 0.25	0.44 ± 0.06	6.14 ± 1.89	1.94 ± 0.58	0.23 ± 0.06	0.01 ± 0.01	0.05 ± 0.02	0.17 ± 0.05
	1	1.86 ± 0.58	0.74 ± 0.12	0.19 ± 0.05	ND	ND	ND	0.13 ± 0.06	0.09 ± 0.03	5.75 ± 0.95	0.77 ± 0.13	0.24 ± 0.04	2.83 ± 0.52	0.39 ± 0.09	0.12 ± 0.02	ND	ND	ND
	3	1.15 ± 0.44	0.37 ± 0.10	ND	ND	ND	ND	ND	ND	2.37 ± 0.47	0.16 ± 0.03	ND	1.02 ± 0.27	0.18 ± 0.04	0.06 ± 0.02	ND	ND	ND
	7	0.58 ± 0.08	ND	ND	ND	ND	ND	ND	ND	0.39 ± 0.10	0.02 ± 0.00	ND	0.56 ± 0.12	0.08 ± 0.01	ND	ND	ND	ND
	14	0.13 ± 0.05	ND	ND	ND	ND	ND	ND	ND	0.26 ± 0.04	ND	ND	0.17 ± 0.03	ND	ND	ND	ND	ND
	21	ND	ND	ND	ND	ND	ND	ND	ND	0.09 ± 0.01	ND	ND	ND	ND	ND	ND	ND	ND
Kidney	0.25	15.36 ± 2.12	6.06 ± 0.97	1.20 ± 0.08	0.11 ± 0.09	0.13 ± 0.04	0.07 ± 0.02	ND	0.27 ± 0.07	38.87 ± 8.00	7.99 ± 1.65	0.78 ± 0.16	3.80 ± 1.55	2.65 ± 0.97	0.37 ± 0.13	ND	0.04 ± 0.02	0.03 ± 0.02
	1	7.24 ± 1.02	2.92 ± 0.53	0.55 ± 0.14	ND	0.05 ± 0.01	0.03 ± 0.01	ND	0.07 ± 0.02	6.47 ± 1.25	1.73 ± 0.33	0.06 ± 0.01	1.13 ± 0.43	0.32 ± 0.12	0.12 ± 0.03	ND	ND	ND
	3	1.15 ± 0.44	0.37 ± 0.10	ND	ND	ND	ND	ND	ND	3.01 ± 0.55	0.82 ± 0.15	ND	0.59 ± 0.11	0.12 ± 0.02	0.02 ± 0.00	ND	ND	ND
	7	0.17 ± 0.02	ND	ND	ND	ND	ND	ND	ND	1.19 ± 0.08	0.38 ± 0.03	ND	0.35 ± 0.08	0.04 ± 0.01	ND	ND	ND	ND
	14	0.04 ± 0.01	ND	ND	ND	ND	ND	ND	ND	0.68 ± 0.08	ND	ND	0.06 ± 0.01	ND	ND	ND	ND	ND
	21	ND	ND	ND	ND	ND	ND	ND	ND	0.09 ± 0.02	ND	ND	ND	ND	ND	ND	ND	ND
Muscle	0.25	0.53 ± 0.22	0.20 ± 0.08	0.06 ± 0.02	ND	ND	ND	ND	ND	1.96 ± 0.53	0.30 ± 0.08	ND	1.02 ± 0.48	0.25 ± 0.10	0.04 ± 0.01	ND	ND	ND
	1	0.33 ± 0.10	0.09 ± 0.02	0.01 ± 0.00	ND	ND	ND	ND	ND	1.69 ± 0.41	0.27 ± 0.07	ND	0.70 ± 0.21	0.14 ± 0.03	ND	ND	ND	ND
	3	0.22 ± 0.03	ND	ND	ND	ND	ND	ND	ND	0.87 ± 0.18	0.10 ± 0.02	ND	0.38 ± 0.09	ND	ND	ND	ND	ND
	7	0.03 ± 0.00	ND	ND	ND	ND	ND	ND	ND	0.41 ± 0.09	ND	ND	0.17 ± 0.04	ND	ND	ND	ND	ND
	14	ND	ND	ND	ND	ND	ND	ND	ND	0.06 ± 0.01	ND	ND	ND	ND	ND	ND	ND	ND
	21	ND	ND	ND	ND	ND	ND	ND	ND	ND	ND	ND	ND	ND	ND	ND	ND	ND
Fat or Skin	0.25	0.78 ± 0.24	0.30 ± 0.10	0.03 ± 0.01	ND	ND	ND	ND	ND	3.04 ± 0.52	0.55 ± 0.09	0.07 ± 0.01	0.29 ± 0.10	0.07 ± 0.02	ND	ND	ND	ND
	1	0.18 ± 0.06	0.06 ± 0.02	ND	ND	ND	ND	ND	ND	2.32 ± 0.67	0.45 ± 0.13	0.05 ± 0.01	0.15 ± 0.03	ND	ND	ND	ND	ND
	3	0.14 ± 0.02	ND	ND	ND	ND	ND	ND	ND	1.33 ± 0.15	0.14 ± 0.02	ND	0.08 ± 0.01	ND	ND	ND	ND	ND
	7	0.06 ± 0.01	ND	ND	ND	ND	ND	ND	ND	0.80 ± 0.16	0.05 ± 0.01	ND	0.03 ± 0.00	ND	ND	ND	ND	ND
	14	ND	ND	ND	ND	ND	ND	ND	ND	0.06 ± 0.01	ND	ND	ND	ND	ND	ND	ND	ND
	21	ND	ND	ND	ND	ND	ND	ND	ND	ND	ND	ND	ND	ND	ND	ND	ND	ND

ND: Data was not detected.

**Table 5:** Summary of the elimination parameters of the total radioactivity and major metabolites in the tissues of swine, broilers, carp and rats.

Species	Tissue	Elimination equation				*t*_1/2_ (d)			
		Total	A0	A1	A2	Total	A0	A1	A2
Swine	Liver	C = 3346e^−0.16t^	C = 2254e^−0.16t^	C = 4369e^−0.67t^	C = 2304e^−0.70t^	4.26	4.38	1.04	0.99
	Kidney	C = 2029e^−0.21t^	C = 1739e^−0.21t^	C = 3514e^−0.77t^	C = 8259e^−1.64t^	3.35	3.37	0.91	0.42
	Muscle	C = 1589e^−0.41t^	C = 1378e^−0.43t^	C = 705e^−0.95t^	−	1.68	1.62	0.73	−
	Fat	C = 673e^−0.20t^	C = 619e^−0.21t^	−	−	3.40	3.37	−	−
Broiler	Liver	C = 2778e^−0.21t^	C = 2226e^−0.20t^	C = 2303e^−0.66t^	−	3.38	3.40	1.05	−
	Kidney	C = 2904e^−0.30t^	C = 2160e^−0.30t^	C = 7939e^−1.02t^	−	2.33	2.31	0.68	−
	Muscle	C = 728e^−0.42t^	C = 598e^−0.41t^	−	−	1.64	1.67	−	−
	Fat	C = 289e^−0.23t^	C = 235e^−0.23t^	−	−	3.00	2.98	−	−
Carp	Liver	C = 953e^−0.10t^	C = 892e^−0.10t^	C = 2161e^−0.64t^	−	6.69	6.63	1.09	−
	Kidney	C = 7461e^−0.19t^	C = 5619e^−0.19t^	C = 2006e^−0.25t^	−	3.56	3.72	2.81	−
	Muscle	C = 1993e^−0.23t^	C = 1953e^−0.24t^	C = 365e^−0.34t^	−	3.03	2.87	2.05	−
	Skin	C = 4433e^−0.29t^	C = 4064e^−0.29t^	C = 226e^−0.35t^	−	2.42	2.40	1.99	−
Rat	Liver	C = 2189e^−0.18t^	C = 1720e^−0.17t^	C = 448e^−0.26t^	C = 222e^−0.43t^	3.96	4.17	2.69	1.61
	Kidney	C = 1529e^−0.21t^	C = 1281e^−0.22t^	C = 393e^−0.33t^	C = 402e^−0.99t^	3.23	3.20	2.11	0.70
	Muscle	C = 1004e^−0.25t^	C = 839e^−0.23t^	−	−	2.83	2.95	−	−
	Fat	C = 192e^−0.26t^	C = 190e^−0.27t^	−	−	2.62	2.59	−	−

No kinetic parameters are obtained because of the terminal phase is less than three time point. The elimination equation is described as follows: C*_t_* = C_0_′e*^-kt^*, where C*_t_* is the concentration at time *t*, C_0_′ is a pre-exponential term (fictitious concentration at *t* = 0) and *k* is the elimination rate constant. The half-life of elimination (*t_1/2_*) is calculated from the equation *t_1/2_* = ln2/*k*.
