# Supplementary material for: Metabolism and Disposition of Aditoprim in Swine, Broilers, Carp and Rats
Source: Sci Rep. 2016 Feb 3;6:20370. doi: 10.1038/srep20370 (PMC4738305; doi:10.1038/srep20370)

Metabolism and Disposition of Aditoprim in Swine, Broilers, Carp and Rats

Liye Wang^a,b,c^, Lingli Huang^b,c^, Yuanhu Pan^b,c^, Kamil Kuča^d,e^, Blanka Klímová^d^, Qinghua Wu^d,f^, Shuyu Xie^a,b^, Ijaz Ahmad^a^, Dongmei Chen^a,c^, Yanfei Tao^a,c^, Dan Wan^g^, Zhenli Liu^a,c^, Zonghui Yuan^a,b,c^*

^a^National Reference Laboratory of Veterinary Drug Residues and MAO Key Laboratory for Detection of Veterinary Drug Residues, ^b^MOA Laboratory for Risk Assessment of Quality and Safety of Livestock and Poultry Products, ^c^Hubei Collaborative Innovation Center for Animal Nutrition and Feed Safety, Huazhong Agricultural University, Wuhan, Hubei 430070, China, ^d^Center for Basic and Applied Research, Faculty of Informatics and Management, University of Hradec Kralove, Hradec Kralove, Czech Republic, ^e^Biomedical research Center, University Hospital Hradec Kralove, Hradec Kralove, Czech Republic, ^f^College of Life Science, Yangtze University, Jingzhou 434025, China, ^g^Hunan Provincial Engineering Research Center for Healthy Livestock and Poultry Production, Key Laboratory of Agro-ecological Processes in Subtropical Region, Institute of Subtropical Agriculture, Chinese Academy of Sciences, Changsha, 410125, China

***Corresponding author: Prof. Zonghui Yuan, D. V. M. & Ph. D,** Phone: 0086-27-8728 7186, Fax: 0086-27-8767 2232, E-mail: [yuan5802@mail.hzau.edu.cn](mailto:yuan5802@mail.hzau.edu.cn)

Table S1: Concentrations (mg/kg) and elimination kinetics of total residues in the tissues of swine following a multi-dose of ^3^H-ADP at 5 mg/kg b. w. for 7 days (n=4).

| Tissues | Concentrations (mg/kg) | | | | | | Elimination kinetics | |
| --- | --- | --- | --- | --- | --- | --- | --- | --- |
|  | 0.25 | 1 | 3 | 7 | 14 | 21 | *K*e | *t*_1/2_ (d) |
| Heart | 3.53±0.16 | 1.51±0.63 | 0.48±0.04 | 0.15±0.04 | 0.02±0.00 | ND | 0.29 | 2.36 |
| Liver | 21.65±2.75 | 6.95±2.02 | 2.37±0.17 | 0.86±0.15 | 0.37±0.03 | ND | 0.16 | 4.26 |
| Spleen | 25.40±2.91 | 6.89±3.81 | 0.91±0.09 | 0.31±0.01 | 0.09±0.01 | ND | 0.20 | 3.40 |
| Lung | 36.92±3.38 | 9.63±4.57 | 0.89±0.03 | 0.23±0.05 | 0.04±0.00 | ND | 0.28 | 2.46 |
| Kidney | 46.83±5.24 | 10.93±5.42 | 1.26±0.10 | 0.38±0.01 | 0.12±0.01 | ND | 0.21 | 3.35 |
| Stomach | 15.32±5.59 | 4.00±2.18 | 0.68±0.16 | 0.15±0.03 | ND | ND | 0.52 | 1.33 |
| Small Intestine | 4.53±1.40 | 1.94±0.63 | 0.26±0.02 | 0.06±0.03 | ND | ND | 0.54 | 1.28 |
| Large Intestine | 6.40±1.26 | 2.16±1.15 | 0.28±0.03 | 0.10±0.03 | ND | ND | 0.47 | 1.47 |
| Bladder | 5.38±0.54 | 2.31±1.39 | 0.60±0.18 | 0.11±0.03 | ND | ND | 0.50 | 1.39 |
| Skin | 3.77±0.75 | 1.89±0.46 | 0.63±0.39 | 0.44±0.10 | 0.04±0.01 | ND | 0.42 | 1.64 |
| Clothing hair | 0.33±0.16 | 0.32±0.14 | 0.30±0.11 | 0.13±0.01 | 0.05±0.01 | ND | 0.17 | 4.16 |
| Fat | 1.63±0.38 | 0.57±0.11 | 0.35±0.04 | 0.17±0.05 | ND | ND | 0.20 | 3.40 |
| Muscle | 2.69±0.27 | 1.10±0.47 | 0.43±0.01 | 0.09±0.02 | ND | ND | 0.41 | 1.68 |
| Thymus | 11.65±0.31 | 3.02±1.54 | 0.73±0.20 | 0.29±0.06 | ND | ND | 0.37 | 1.88 |
| Pancreas | 16.20±2.32 | 5.06±2.61 | 0.74±0.05 | 0.24±0.07 | 0.04±0.00 | ND | 0.27 | 2.60 |
| Adrenal | 48.60±3.20 | 13.85±7.65 | 1.33±0.13 | 0.29±0.05 | 0.06±0.00 | ND | 0.28 | 2.49 |
| Lymph | 16.96±0.33 | 4.74±2.60 | 0.84±0.10 | 0.25±0.04 | ND | ND | 0.46 | 1.50 |
| Blood | 2.21±0.21 | 1.57±0.28 | 0.62±0.02 | 0.09±0.02 | ND | ND | 0.48 | 1.44 |
| Bile | 66.00±30.11 | 19.60±10.41 | 1.60±0.10 | 0.22±0.01 | ND | ND | 0.71 | 0.97 |

ND: Data was not detected.

Table S2: Concentrations (mg/kg) and elimination kinetics of total residues in the tissues of broilers following a multi-dose of ^3^H-ADP at 5 mg/kg b. w. for 7 days (n=6).

| Tissues | Concentrations (mg/kg) | | | | | | Elimination kinetics | |
| --- | --- | --- | --- | --- | --- | --- | --- | --- |
|  | 0.25 | 1 | 3 | 7 | 14 | 21 | *K*e | *t*_1/2_ (d) |
| Heart | 2.27±0.52 | 0.81±0.25 | 0.23±0.03 | 0.08±0.00 | 0.01±0.00 | ND | 0.29 | 2.41 |
| Liver | 13.31±1.98 | 3.05±0.66 | 1.55±0.21 | 0.63±0.03 | 0.16±0.02 | ND | 0.21 | 3.38 |
| Spleen | 14.03±3.12 | 3.08±0.51 | 0.62±0.02 | 0.10±0.01 | 0.03±0.02 | ND | 0.26 | 2.63 |
| Lung | 11.41±1.91 | 3.27±0.25 | 0.59±0.05 | 0.08±0.00 | ND | ND | 0.61 | 1.14 |
| Kidney | 23.54±3.83 | 11.04±2.65 | 1.70±0.28 | 0.21±0.00 | 0.06±0.00 | ND | 0.30 | 2.33 |
| Maw | 7.77±1.06 | 1.46±0.64 | 0.25±0.03 | 0.05±0.01 | ND | ND | 0.53 | 1.31 |
| Muscular stomach | 1.92±0.57 | 0.69±0.10 | 0.25±0.03 | 0.04±0.01 | ND | ND | 0.46 | 1.52 |
| Glandular stomach | 6.93±1.51 | 1.54±0.36 | 0.27±0.05 | 0.04±0.00 | ND | ND | 0.59 | 1.17 |
| Large Intestine | 6.29±1.45 | 1.25±0.23 | 0.12±0.03 | 0.01±0.00 | ND | ND | 0.90 | 0.77 |
| Small Intestine | 8.23±0.93 | 4.06±1.32 | 0.29±0.04 | 0.04±0.00 | ND | ND | 0.75 | 0.92 |
| Feathers | 0.85±0.17 | 0.53±0.12 | 0.27±0.03 | 0.10±0.01 | ND | ND | 0.28 | 2.50 |
| Skin | 1.78±0.42 | 0.74±0.11 | 0.25±0.02 | 0.05±0.01 | ND | ND | 0.43 | 1.61 |
| Fat | 0.78±0.32 | 0.43±0.05 | 0.24±0.02 | 0.04±0.01 | ND | ND | 0.42 | 1.64 |
| Muscle | 1.10±0.44 | 0.23±0.10 | 0.14±0.02 | 0.06±0.01 | ND | ND | 0.23 | 3.00 |
| Pancreas | 11.41±3.46 | 2.55±0.56 | 0.49±0.04 | 0.07±0.01 | ND | ND | 0.57 | 1.21 |
| Bursa of Fabricius | 9.62±1.43 | 4.20±0.86 | 0.51±0.09 | 0.10±0.01 | 0.01±0.00 | ND | 0.33 | 2.11 |
| Brain | 2.07±0.54 | 1.02±0.28 | 0.44±0.05 | 0.06±0.01 | ND | ND | 0.47 | 1.48 |
| Bile | 25.15±8.55 | 11.46±1.16 | 3.96±1.06 | 0.25±0.08 | ND | ND | 0.71 | 1.08 |
| Blood | 0.77±0.16 | 0.42±0.06 | 0.16±0.03 | 0.06±0.01 | ND | ND | 0.31 | 2.25 |

ND: Data was not detected.

Table S3: Concentrations (mg/kg) and elimination kinetics of total residues in the tissues of carp following a multi-dose of ^3^H-ADP at 5 mg/kg b. w. for 7 days (n=6).

| Tissues | Concentrations (mg/kg) | | | | | | Elimination kinetics | |
| --- | --- | --- | --- | --- | --- | --- | --- | --- |
|  | 0.25 | 1 | 3 | 7 | 14 | 21 | *K*e | *t*_1/2_ (d) |
| Heart | 5.37±0.50 | 2.58±0.57 | 1.11±0.08 | 0.34±0.05 | 0.14±0.02 | 0.01±0.00 | 0.30 | 2.33 |
| Liver | 15.59±1.52 | 6.76±1.12 | 2.53±0.21 | 0.40±0.08 | 0.30±0.05 | 0.09±0.01 | 0.10 | 6.69 |
| Spleen | 63.23±2.89 | 27.12±4.89 | 8.17±0.59 | 0.73±0.17 | 0.25±0.05 | 0.03±0.00 | 0.23 | 3.04 |
| Branchia | 12.73±1.93 | 5.46±0.84 | 1.66±0.08 | 0.22±0.04 | 0.05±0.00 | 0.01±0.00 | 0.23 | 3.03 |
| Kidney | 47.64±9.81 | 8.30±1.02 | 3.83±0.39 | 1.57±0.11 | 0.72±0.10 | 0.10±0.02 | 0.19 | 3.56 |
| Intestine | 1.12±2.09 | 5.35±0.51 | 1.85±0.25 | 0.42±0.09 | 0.16±0.06 | ND | 0.21 | 3.26 |
| Muscle | 2.26±0.62 | 1.96±0.48 | 0.96±0.10 | 0.43±0.09 | 0.08±0.00 | ND | 0.23 | 3.03 |
| Skin | 3.66±0.62 | 2.82±0.91 | 1.48±0.16 | 0.87±0.17 | 0.07±0.01 | ND | 0.29 | 2.42 |
| Roe | 4.74±1.03 | 3.86±0.76 | 2.24±0.36 | 0.85±0.40 | 0.47±0.13 | 0.09±0.01 | 0.16 | 4.30 |
| Scales | 2.59±0.32 | 2.01±0.45 | 0.80±0.04 | 0.29±0.07 | 0.06±0.01 | ND | 0.24 | 2.87 |
| Colla piscis | 2.78±0.76 | 1.50±0.22 | 0.41±0.04 | 0.10±0.02 | 0.03±0.01 | ND | 0.24 | 2.91 |
| Bile | 334.60±69.02 | 89.59±3.87 | 24.33±1.91 | 1.34±0.29 | 0.13±0.05 | 0.01±0.00 | 0.38 | 1.83 |
| Blood | 1.73±0.18 | 1.06±0.10 | 0.40±0.08 | 0.19±0.02 | 0.05±0.01 | ND | 0.20 | 3.47 |

ND: Data was not detected.

Table S4: Concentrations and elimination kinetics of total residues in the tissues of rats following a multi-dose of ^3^H-ADP at 5 mg/kg b. w. for 7 days (n=6).

| Tissues | Concentrations (mg/kg) | | | | | | Elimination kinetics | |
| --- | --- | --- | --- | --- | --- | --- | --- | --- |
|  | 0.25 | 1 | 3 | 7 | 14 | 21 | *K*e | *t*_1/2_ (d) |
| Heart | 1.55±0.15 | 0.92±0.12 | 0.51±0.05 | 0.19±0.09 | 0.04±0.00 | ND | 0.24 | 2.95 |
| Liver | 8.69±1.40 | 3.41±0.63 | 1.25±0.34 | 0.68±0.09 | 0.19±0.03 | ND | 0.18 | 3.96 |
| Spleen | 6.24±2.31 | 2.08±0.86 | 0.80±0.17 | 0.50±0.05 | 0.06±0.00 | ND | 0.24 | 2.89 |
| Lung | 4.43±0.82 | 1.19±0.32 | 0.50±0.06 | 0.26±0.05 | ND | ND | 0.24 | 2.88 |
| Kidney | 6.99±1.74 | 1.58±0.39 | 0.73±0.08 | 0.40±0.05 | 0.07±0.00 | ND | 0.21 | 3.23 |
| Stomach | 5.25±1.98 | 0.74±0.27 | 0.23±0.05 | 0.14±0.03 | ND | ND | 0.27 | 2.61 |
| Small Intestine | 7.67±3.35 | 0.54±0.18 | 0.13±0.05 | 0.05±0.01 | ND | ND | 0.36 | 1.93 |
| Large Intestine | 2.52±0.95 | 0.30±0.10 | 0.14±0.04 | 0.03±0.01 | ND | ND | 0.40 | 1.72 |
| Gonades | 3.12±0.87 | 1.90±0.89 | 0.43±0.13 | 0.15±0.04 | ND | ND | 0.40 | 1.73 |
| Bladder | 4.04±2.96 | 1.19±0.36 | 0.32±0.08 | 0.04±0.02 | ND | ND | 0.56 | 1.23 |
| Skin | 1.60±0.19 | 1.17±0.36 | 0.69±0.07 | 0.36±0.08 | 0.04±0.00 | ND | 0.19 | 3.59 |
| Fat | 0.36±0.18 | 0.16±0.09 | 0.08±0.04 | 0.03±0.01 | ND | ND | 0.26 | 2.62 |
| Muscle | 1.31±0.19 | 0.86±0.14 | 0.42±0.01 | 0.19±0.08 | ND | ND | 0.25 | 2.83 |
| Brain | 0.97±0.18 | 0.78±0.16 | 0.45±0.06 | 0.17±0.11 | ND | ND | 0.26 | 2.69 |
| Thymus | 3.87±0.90 | 1.47±0.59 | 0.42±0.05 | 0.20±0.06 | 0.01±0.01 | ND | 0.35 | 2.01 |
| Pancreas | 3.14±0.61 | 1.00±0.41 | 0.50±0.05 | 0.20±0.04 | 0.02±0.00 | ND | 0.26 | 2.65 |
| Adrenal | 6.06±3.09 | 1.00±0.36 | 0.42±0.04 | 0.17±0.02 | ND | ND | 0.28 | 2.46 |
| Blood | 1.15±0.42 | 0.92±0.25 | 0.59±0.05 | 0.34±0.06 | ND | ND | 0.22 | 3.14 |

ND: Data was not detected.

Table S5: Average percentage (%) of ADP and its metabolites in total dose after a single oral administration of a 5 mg/kg b. w ^3^H-ADP to swine, broilers, carp, and rats.

| Animals | Samples | Compounds | | | | | | | | | | | | |
| --- | --- | --- | --- | --- | --- | --- | --- | --- | --- | --- | --- | --- | --- | --- |
|  |  | A0 | A1 | A2 | A3 | A4 | A5 | A6 | A7 | A8 | A9 | A10 | A11 | A12 |
| Swine | Urine | 33.4 | 13.3 | 11.3 | 5.4 | 3.1 | 1.9 | 3.7 | 1.8 | 1.0 | 0.5 | 2.4 | 0.7 | - |
|  | Feces | 16.0 | 2.9 | 0.5 | - | - | - | - | - | - | - | - | - | - |
| Broilers | Excreta | 64.3 | 13.7 | 4.8 | 6.4 | - | - | - | - | - | 0.2 | 0.2 | 0.3 | 5.0 |
| Carp | Excreta | 82.4 | 8.9 | 2.9 | - | - | - | - | - | - | - | - | - | - |
| Rat | Urine | 61.3 | 13.8 | 1.1 | - | - | - | 0.5 | - | - | 3.1 | 1.1 | - | - |
|  | Feces | 14.0 | 2.7 | 0.7 | - | - | - | - | - | - | - | - | - | - |

ND: Data was not detected.

Figure S1: Accurate MS spectra ([M+H]^+^) of ADP (A0) and its metabolites (A1–A12).


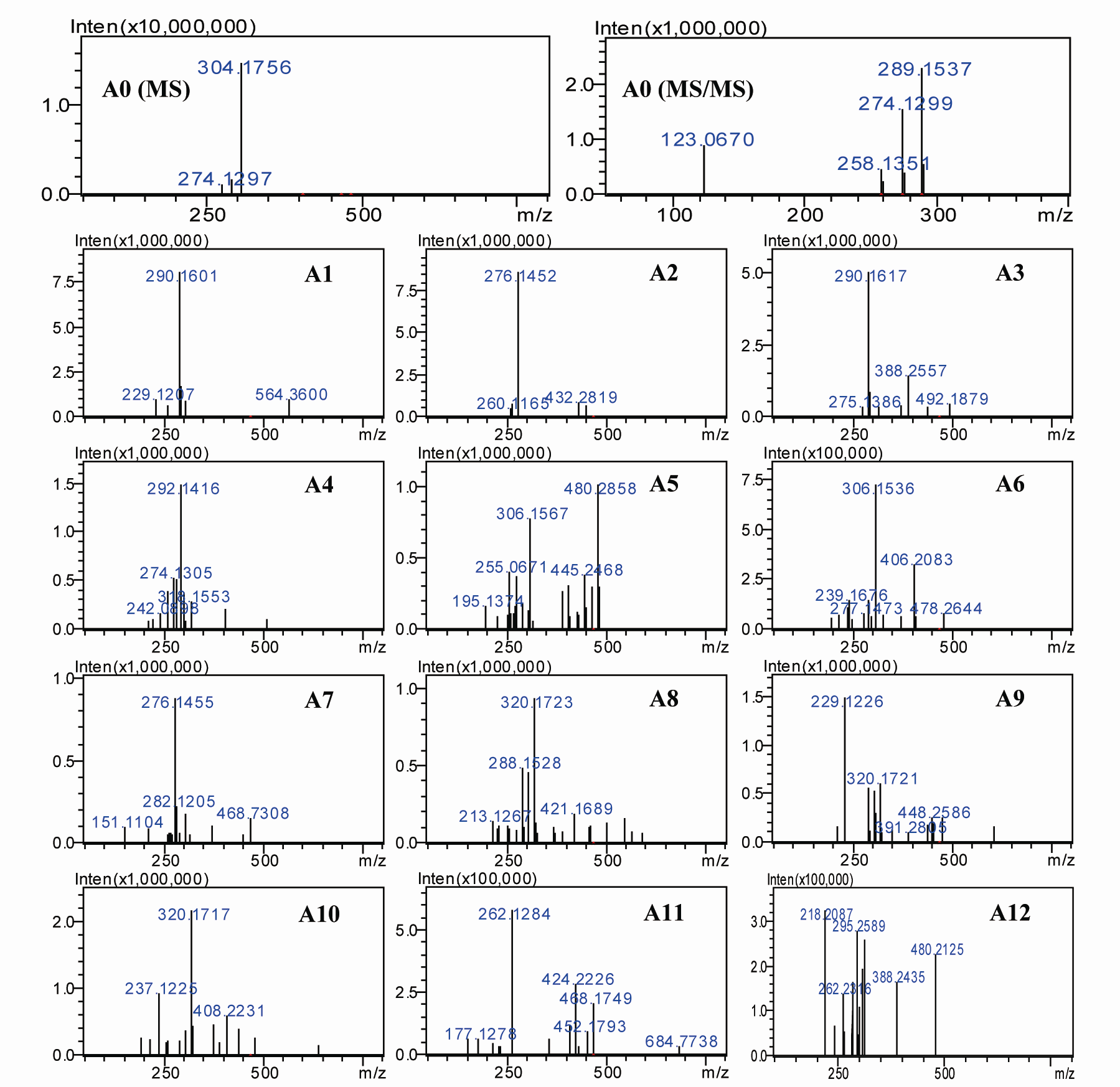


Figure S2: The elimination curves of total radioactivity and individual radio-compounds in the major tissues of swine, broilers, carp and rats following a multi-dose of ^3^H-ADP at 5 mg/kg b. w. 7 days.


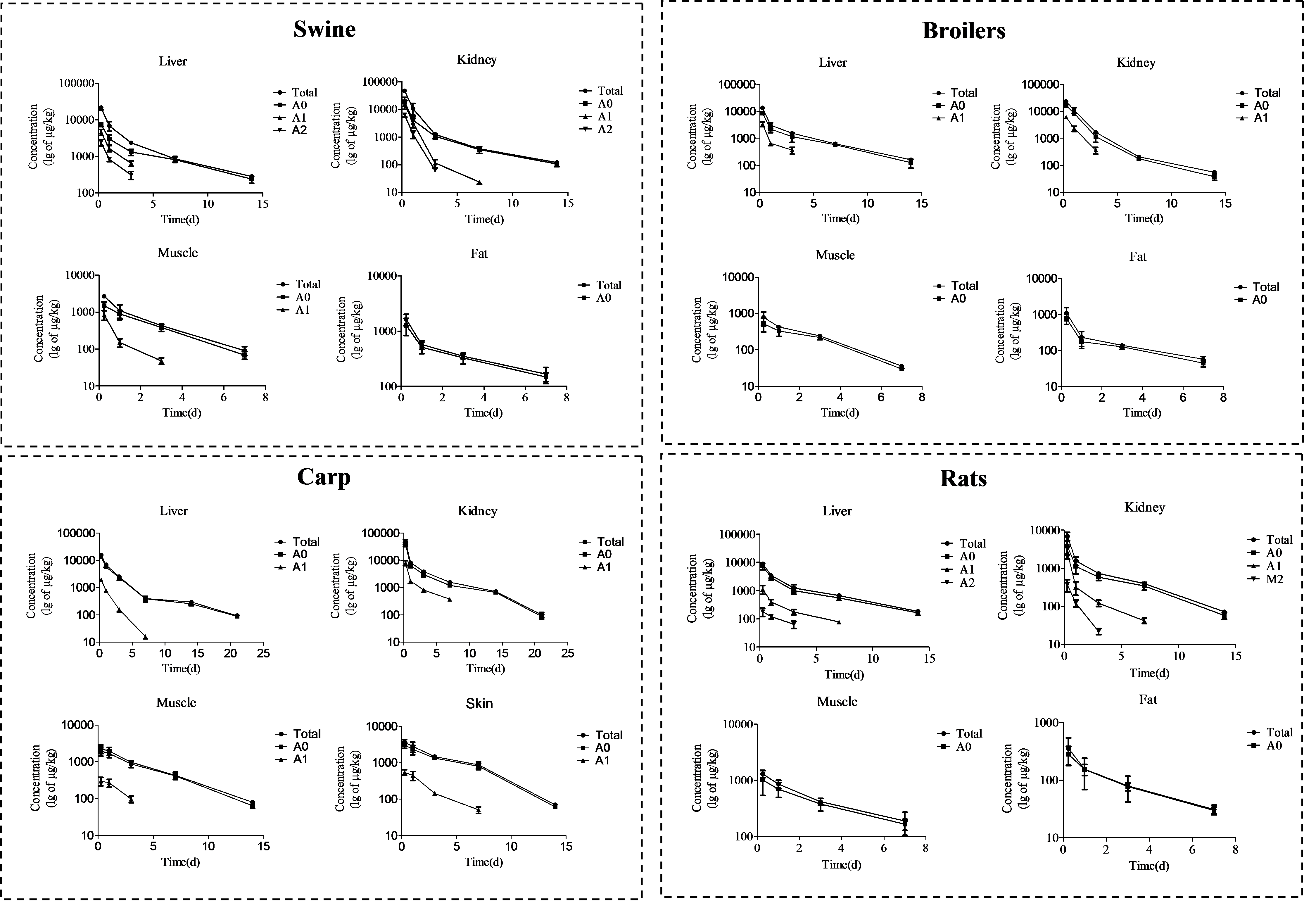


Figure S3: Synthetic route of ^3^H-ADP: (i) 1.3 equiv NaBH_4_, methanol, room temperature, 30min; (ii) 1.0 equiv 40% HBr, 1.530% H_2_O_2_, dichloromethane, 5–10 °C, 5 h; (iii) Swern oxidation, CH_2_Cl_2_, −80 °C, 1h, (iv) 2 equiv 3-methoxypropionitrole, sodium methoxide, methanol, 30 °C, 2h; (v) 2 equiv guanidine carbonate, sodium ethylate, absolute ethyl alcohol, reﬂux, 5 h; (vi) 250 mmHg tritium gas, 10% Pd/C, methanol–DMF, 60 °C, 2 h.


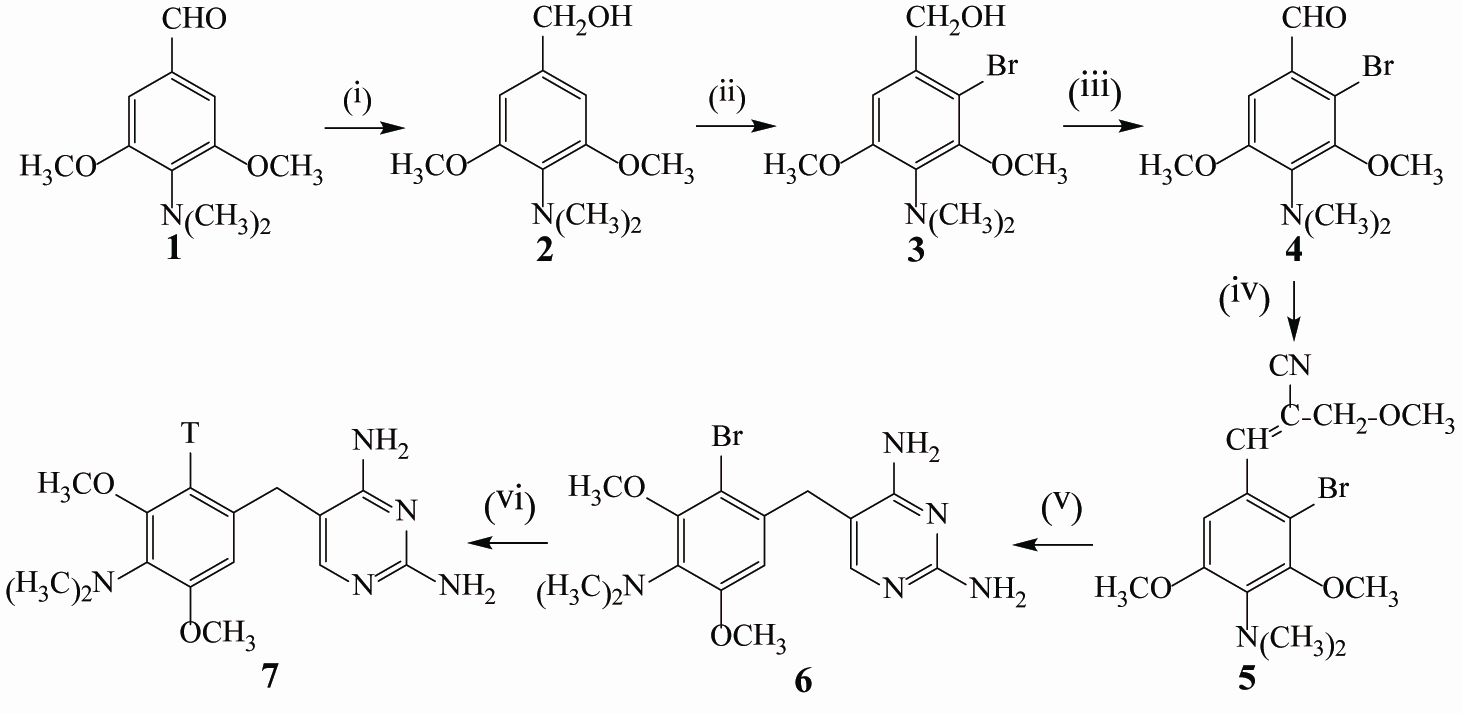

Supplement: Supplementary Information [file srep20370-s1.doc]
